# Gut microbial dysbiosis in patients with Cushing’s disease in long-term remission. Relationship with cardiometabolic risk

**DOI:** 10.3389/fendo.2023.1074757

**Published:** 2023-06-05

**Authors:** Elena Valassi, Chaysavanh Manichanh, Vincent Amodru, Pedro González Fernández, Sonia Gaztambide, Francisca Yañez, Luciana Martel-Duguech, Manel Puig-Domingo, Susan M. Webb

**Affiliations:** ^1^ Endocrinology and Nutrition Department, Germans Trias i Pujol Hospital and Research Institute, Badalona, Spain; ^2^ School of Medicine, Universitat Internacional de Catalunya (UIC), Barcelona, Spain; ^3^ Centro de Investigación Biomédica en Red de Enfermedades Raras (CIBERER), Unit 747, ISCIII, Barcelona, Spain; ^4^ Microbiome Group, Vall d’Hebron Institut de Recerca (VHIR), Vall d’Hebron Hospital, Barcelona, Spain; ^5^ Endocrinology Department, Cruces University Hospital, Bilbao, Spain; ^6^ Biocruces Bizkaia, UPVEHU, CIBERDEM, Endo-ERN, SpainCruces Hospital, Bilbao, Spain; ^7^ IIB-Sant Pau and Department of Endocrinology, Hospital Sant Pau, Barcelona, Spain; ^8^ Department of Medicine, Universitat Autònoma de Barcelona, Barcelona, Spain

**Keywords:** Cushing's syndrome, Cushing's disease, gut microbiota, cardiovascular risk

## Abstract

**Background:**

Patients with Cushing’s disease (CD) in remission maintain an increased cardiovascular risk. Impaired characteristics of gut microbiome (dysbiosis) have been associated with several cardiometabolic risk factors.

**Methods:**

Twenty-eight female non-diabetic patients with CD in remission with a mean ± SD) age of 51 ± 9 years, mean ( ± SD) BMI, 26 ± 4, median (IQR) duration of remission, 11(4) years and 24 gender-, age, BMI–matched controls were included. The V4 region of the bacterial 16S rDNA was PCR amplified and sequenced to analyse microbial alpha diversity (Chao 1 index, observed number of species, Shannon index) and beta diversity analysis through the Principal Coordinates Analysis (PCoA) of weighted and unweighted UniFrac distances. Inter-group difference in microbiome composition was analysed using MaAsLin2.

**Results:**

The Chao 1 index was lower in CD as compared with controls (Kruskal-Wallis test, q = 0.002), indicating lower microbial richness in the former. Beta diversity analysis showed that faecal samples from CS patients clustered together and separated from the controls (Adonis test, p<0.05). *Collinsella*, a genus form of the Actinobacteria phylum was present in CD patients only, whereas *Sutterella*, a genus from *Proteobacteria phylum*, was scarcely detectable/undetectable in CD patients as well as *Lachnospira*, a genus of the *Lachnospiraceae* family of the *Firmicutes* phylum. In CS, the Chao 1 index was associated with fibrinogen levels and inversely correlated with both triglyceride concentrations and the HOMA-IR index (p<0.05).

**Conclusions:**

Patients with CS in remission have gut microbial dysbiosis which may be one of the mechanisms whereby cardiometabolic dysfunctions persist after “cure”.

## Introduction

Chronic endogenous hypercortisolism in Cushing’s syndrome (CS) is associated with several cognitive, musculoskeletal and cardiometabolic alterations, which negatively impact quality of life and survival (1, 2). It is well documented that some of the systemic comorbidities which characterize the active phase of CS, such as visceral obesity, dyslipidemia, insulin-resistance, atherosclerosis and hypercoagulability, do not fully revert after successful treatment of hypercortisolism and may persist in the long-term (3, 4). As a matter of fact, mortality, mainly due to cardiovascular diseases, is increased in “cured” CS patients, with concomitant type 2 diabetes and hypertension being one of the most important risk factors affecting longevity (5, 6). The pathogenic mechanisms underlying this residual metabolic morbidity is multiple and not fully elucidated.

The gut microbiota (GM) comprises the trillions of microorganisms living in the human gastrointestinal tract (7, 8). The GM performs essential functions to maintain human homeostasis, such as harvesting energy, digestion of complex host-indigestible polysaccharides, protection against pathogens, and modulation of host immunity (9). Moreover, the GM releases several molecules into the gut lumen, including metabolites (e.g., short-chain fatty acids, SCFAs) and components of the bacterial cell wall (e.g., lipopolysaccharide, LPS), which cross the epithelial barrier and reach distal targets *via* the bloodstream (10). Thus, the GM interacts with several organs and tissues, including the brain, the liver, and the adipose tissue, directly modulating their physiology, metabolic functions, and immune reactions (11).

Several environmental and biological factors can cause a dysbiosis of the gut microbiome, defined as the imbalance in commensal microbiome and outgrowth of pathogenic bacteria along with the loss of microbial diversity (12). In the last two decades, a tremendous body of evidence has demonstrated a mechanistic involvement of gut dysbiosis in the etiopathogenesis of multiple systemic diseases, including the constellation of risk factors which are associated with the metabolic syndrome, such as obesity, dyslipidemia and cardiovascular disease (9, 11, 12).

Our study was aimed at evaluating the characteristics of GM in female patients with Cushing’s disease (CD) in long-term remission as compared with gender-, age-, BMI-, -matched healthy controls, and investigate if there is a relationship between GM richness and both body composition indexes and cardiometabolic risk factors in CD patients.

## Methods

### Patients

**Figure 1:**
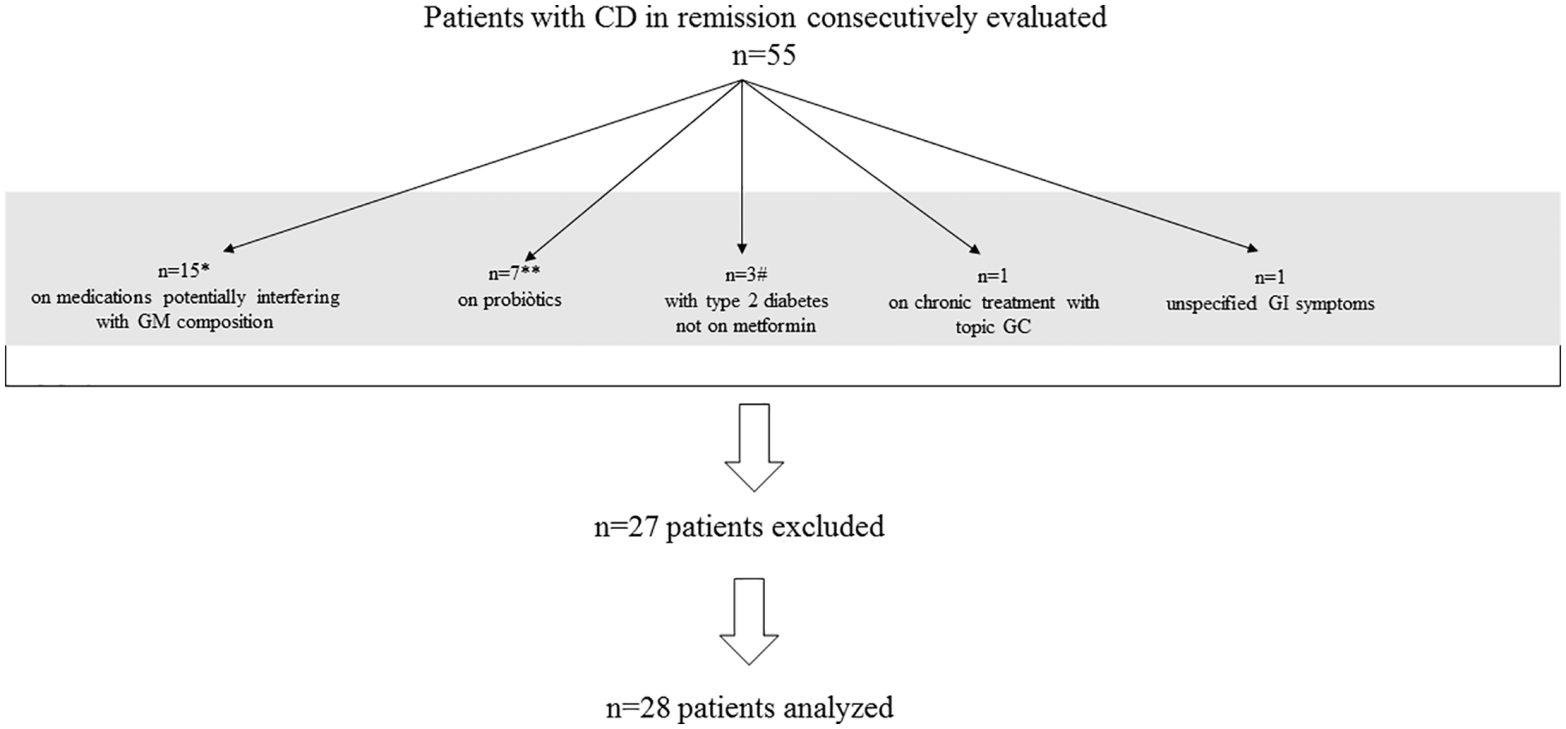
Patient recruitment flow-chart. CD, Cushing’s disease; GM, gut microbiome; GC, glucocorticoids; GI, gastrointestinal The gray bar includes 27 excluded patients; *Of 15 patients, 9 were on proton-pomp inhibitors; 8 on metformin and 6 on antidepressannts/anxiolytics; some patients were taking more than one of different classes; **Of 10 patients taking propbiotics, three were also on treatment with some medications potentially interfering with GM composition and, therefore, included in the corresponding group of 15 patients; # patients with type 2 diabetes were 9, of whom 6 on metformin and, therefore, included in the group of 15 patients on medications potentially interfering with GM composition.

Fifty-five patients with Cushing’s disease (CD) below the age of 60 years, who had been in remission for a minimum of 5 years, were consecutively recruited during their follow-up visits at the endocrine clinic of the Hospital Sant Pau, Barcelona (Spain) (n=40) and at the endocrine clinic of the Hospital Cruces, Bilbao (Spain) (n=15) (**Figure 1**). They were asked to fill out a questionnaire assessing their dietary habits and gastrointestinal health, which was specifically designed to select candidate subjects for the study. In particular, patients and controls were excluded if they had taken probiotics/antibiotics in the previous three months; were on treatment with proton-pump inhibitors, metformin and antidepressants/anxiolytics medications; had irritable bowel syndrome, inflammatory bowel disease, or coeliac disease; had complained of gastrointestinal symptoms in the previous month; received extreme diet intervention such as low fermentable oligo-, di-, mono-saccharides and polyols (FODMAP) diet. Other exclusion criteria were: active disease, treatment with local or systemic glucocorticoids during the previous year, kidney or liver dysfunction, type 2 diabetes mellitus (**Figure 1**).

Finally, 28 female patients (25 from the Hospital Sant Pau and 3 from the Hospital Cruces) were included in the study and matched with 24 BMI- and age-matched unrelated female workers of our institution who complied with the same selection criteria applied to the patients (**Figure 1**).

Diagnosis of CS was made after clinical, biochemical, and radiological evaluations, based on internationally agreed guidelines (13). All patients had abnormal values on at least two of the following tests: elevated UFC, late-night salivary or serum cortisol, 1 mg overnight dexamethasone suppression test (ODST), or 48-hour 2 mg/day low-dose dexamethasone suppression test (LDDST).

Twenty-eight patients had CD due to a microadenoma (*n* = 25) or a macroadenoma (*n* = 3). The median duration of hypercortisolism was 27 (22) months and was defined as the time elapsed from the initial symptoms, as referred by patients, and final diagnosis of CS. Twenty-one patients (75%) received preoperative treatment with steroidogenesis inhibitors to control clinical symptoms of hypercortisolism. All the CD patients underwent transsphenoidal surgery (TSS) a median of 166 (124) months previously, and 7 of them (25%) also received radiotherapy a median of 142 (108) months after unsuccessful surgery (*n* = 1) or relapse (*n* = 6). Mean ( ± SD) time of remission, defined as the time elapsed from diagnostic confirmation of remission to study entry, was 14 ± 6 years, [median, 13 (6); and range, 12 to 216 months]. CD was considered in remission if either adrenal insufficiency was demonstrated (basal morning cortisol <171 nmol/L [<6.2μg/dL] and/or undetectable 24-hour free urinary cortisol) or morning cortisol suppression <50nmol/L (<1.8μg/dL) after 1 mg dexamethasone overnight was observed. Twenty-four (86%) had received hydrocortisone (HC) replacement (between 10 and 20 mg per day) for a median of 19 (21) months after surgery. Median time free from HC replacement was 96 (102) months. At study entry, no patients were on HC replacement and were re-evaluated for possible pituitary insufficiency. One patient had growth hormone deficiency (GHD) and was replaced with recombinant human GH (mean duration of treatment [± SD] 78 ± 34 months). Twenty-four patients (86%) and 20 controls (83%) were postmenopausal. The mean (± SD) duration of menopause was 104 ± 56 months in patients and 87 ± 61 months in controls. No patients were taking oestrogen/progesterone hormone replacement at the study entry. No patients with pituitary-dependent CS developed gonadotropin deficiency after surgery. Five (18%) were hypothyroid (three due to TSH deficiency; two due to primary hypothyroidism, of whom, one associated with Hashimoto’s thyroiditis and one as a consequence of previous thyroid lobectomy) and all of them were on stable doses (65 ± 22 μg/day) of L-thyroxine replacement (mean duration of treatment [ ± SD] 172 ± 36 months).

The degree of physical activity was assessed using the International Physical Activity Questionnaire (IPAQ), as elsewhere described (14, 15). Twenty-four patients and twenty-two controls were classified as having low physical activity level, 4 patients and two controls as having moderate physical activity level.

No CD patients were smokers whereas two controls smoke between 5 and 10 cigarettes per day. All patients and controls declared to be social drinker.

All subjects gave written informed consent to participate in this study, which was approved by the local Ethical Committee of the Hospital Sant Pau (CEIC, Barcelona, Spain). Information about group size, age, gender, and BMI is given in **Table 1**.

**Table 1:** Comparison of cardiometabolic parameters between 28 female patients with Cushing’s syndrome (CS) and 24 female age- and BMI-matched controls.

Parameter	Cushing’s patients (n=28)	Controls (n=24)	P value
Age	51 ± 9	49 ± 8	0.88
BMI (Kg/m^2^)	26 ± 4	26 ± 3	0.91
Waist (cm)	95 ± 11	92 ± 12	0.43
Body mass (Kg)	68.6 ± 12.5	67.4 ± 10.7	0.70
Total fat mass (Kg)	29.2 ± 7.8	26.3 ± 8.6	0.25
Trunk fat mass (%)	43.7 ± 7	37.3 ± 9.9	**0.021**
Total lean mass (Kg)	33 ± 8.8	34.4 ± 4.8	0.40
Glycemia (mg/dl)	93 ± 19	91 ± 9	0.51
Insulin (pmol/L)	76 ± 42	63.5 ± 37	0.063
HOMA-IR	6.5 ± 2.7	2.8 ± 2.7	0.052
HbA1C (%)	5.6 ± 0.6	5.4 ± 0.4	**0.046**
Total cholesterol (mg/dl)	210 ± 30	216 ± 39	0.52
HDL-c (mg/dl)	53 ± 11	63 ± 13	**0.043**
LDL-c (mg/dl)	134 ± 36	122 ± 35	0.43
Triglycerides (mg/dl)	132 ± 47	101 ± 75	**0.038**
Systolic BP	127 ± 22	121 ± 18	0.30
Diastolic BP	80 ± 12	75 ± 11	0.14
Fibrinogen (g/L)	3 ± 1	3 ± 2	**0.040**

BMI, Body Mass Index; data were available in all patients. The bold values indicates p< 0.05.

### Samples and sequences

Twenty-eight faecal samples were processed for genomic DNA extraction, which was used to PCR amplify the V4 region of the 16S rDNA gene as previously described (16). The amplicons were purified using QIAquick PCR Purification Kit (Qiagen, Barcelona, Spain). The pooled amplicons (2 nM) were then subjected to sequencing using Illumina MiSeq technology at the technical support unit of the Autonomous University of Barcelona (UAB, Spain), following standard Illumina platform protocols.

### Sequence analysis

Analysis of raw sequence data was performed using QIIME2 (17), a microbiome bioinformatics platform (18). We compared 28 CS patients with 24 age-, gender, BMI- matched controls healthy controls. A total of 1.68 million of sequences from patients and healthy controls were demultiplexed to attribute sequence reads to the appropriate samples. The sequence reads were then denoised and dereplicated into amplicon sequence variants (ASVs) using the dada2 tool that also filtered out chimeras. Each sequence reads were trimmed using a length of 285 bp. A feature table was generated for all samples with more than 13253 sequences per sample. One of the samples was excluded from further analysis for presenting low sequence reads. The feature table was then used to perform taxonomic classification, alpha and beta diversity analyses, and differential abundance measurements in different experimental groups. Taxonomy was assigned to each ASV using a database that combined both the Greengenes (version 13.8) and the PATRIC databases. Alpha-diversity was analysed using Chao1 and Shannon indices (19, 20) The Chao1 index is a richness estimator, which gives weight to the low-abundant bacterial species as it only accounts for singletons and doubletons. The Shannon index accounts for both the abundance and evenness of the species present and therefore, it gives weight to high-abundant species. Beta-diversity analysis, which measures dissimilarity between sample pair, was performed using a PERMANOVA test (Adonis function in vegan). Differential abundance analysis of the taxonomic profiles was performed using the R version of the MaAsLin2 tool (21), which implements linear mixed-effects models that are useful for multivariable association discovery in population-scale microbiome studies.

### Deposition of sequence data

Sequence data have been deposited in the NCBI database with the following access number: PRJNA891713.

### Body composition assessment and analytical determinations

Total body mass (kg), total fat (%), trunk fat (Kg) and lean mass (Kg) were measured using fan-beamed dual-energy X-ray absorptiometry (DXA) (Hologic Discovery W, Software Apex Version 13.4) under a standardized protocol.

Blood samples were collected after an overnight fast. Routine serum determinations were performed by standard automated techniques: total cholesterol and triglycerides by enzymatic methods, HDL-c by direct method, LDL-c by the Friedewald formula. Serum glucose levels were measured in duplicate by the glucose oxidase method with a Beckman Glucose Analyzer 2 (Brea, CA). The coefficient of variation (CV) was 1.9%. Serum insulin was measured in duplicate by RIA (Medgenix Diagnostics, Fleunes, Belgium). The intra-assay coefficient of variation was 5.2% at a concentration of 10 mU/L and 3.4% at 130 mU/L. The interassay coefficients of variation were 6.9 and 4.5% at 14 and 89 mU/L, respectively. The HOMA-IR index was calculated according to the formula: fasting glucose in mmol/l*fasting insulin in μU/ml/22.5. Glycosylated haemoglobin (HbA_1c_) was measured by the high-performance liquid chromatography method (Bio-Rad, Muenchen, Germany, and autoanalyzer Jokoh HS-10, respectively). Intra- and interassay coefficients of variation were <4% for all these tests. Fibrinogen was measured using the Clauss assay which was performed on a fully automated coagulation analyzer (Sysmex CS-5100 system, Siemens Healthcare Diagnostics, Breda, the Netherlands); intra- and interassay coefficients of variation were <5%.

### Statistical analysis

To assess differences in the microbial alpha diversity and load between groups, we used the non-parametric statistical Wilcoxon signed rank test (pairwise paired comparisons) and Mann-Whitney U test (pairwise unpaired comparisons).

To test for differences in alpha diversity, we used the alpha group significance plugin of QIIME 2. To assess differences in the microbiome composition between groups based on treatment and/or time point, we used the MaAsLin2 (Microbiome Multivariable Association with Linear Models) tool [results were considered significant if FDR (false discovery rate) < 0.05, or q < 0.05)].

Statistical significance of observed differences in cardiometabolic parameters between sample groups was measured by the t-test, with P-values < 0.05 considered significant for all tests. Non-parametric Spearman’s correlation coefficient was used to calculate possible relationships among microbial genera and cardiometabolic parameters.

## Results

### Cardiometabolic parameters

As shown in **Table 1**, CD patients had greater trunk fat mass (%) as compared with controls (43.7 ± 7 vs. 37.3 ± 9.9; p=0.021). In comparison to controls, CD patients also had higher glycosylated haemoglobin (HbA_1c_) (5.6 ± 0.6 vs. 5.4 ± 0.4, p=0.046), higher triglyceride concentrations (132 ± 47 vs. 101 ± 75, p=0.038), lower HDL-c concentrations (53 ± 11 vs. 63 ± 13, p=0.043) and higher fibrinogen concentrations (3 ± 1 vs. 3 ± 2, p=0.040).

### Dysbiosis in Cushing’s disease patients

**Figure 2:**
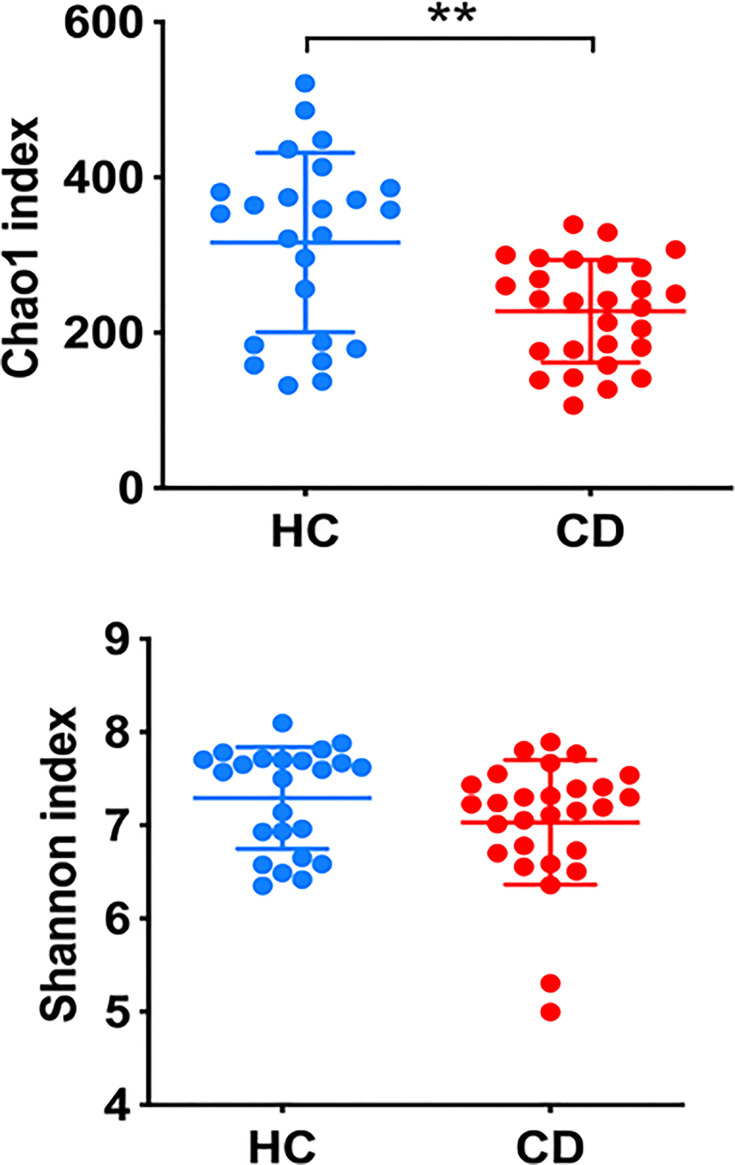
Alpha-diversity as assessed by Chao1 and Shannon indexes. The Chao1 index gives weight to the low-abundant bacterial species, while the Shannon index gives weight to high-abundant species. Kruskal-Wallis test was applied to compare the diversity of healthy controls (HC) and Cushing disease patients, **q < 0.005.

Alpha diversity analysis, as assessed using the Chao1 and Shannon indexes, showed that CD patients presented lower diversity than controls (mean of 300 and 227 for healthy controls and CD, respectively, Kruskal-Wallis test, q = 0.002) based on the Chao1 index but not the Shannon index (mean of 7.3 and 7.0 for HCs and CD, respectively, q = 0.157). The results indicate that patients with CD presented lower GM richness and a trend towards a lower evenness than controls (**Figure 2**).

**Figure 3:**
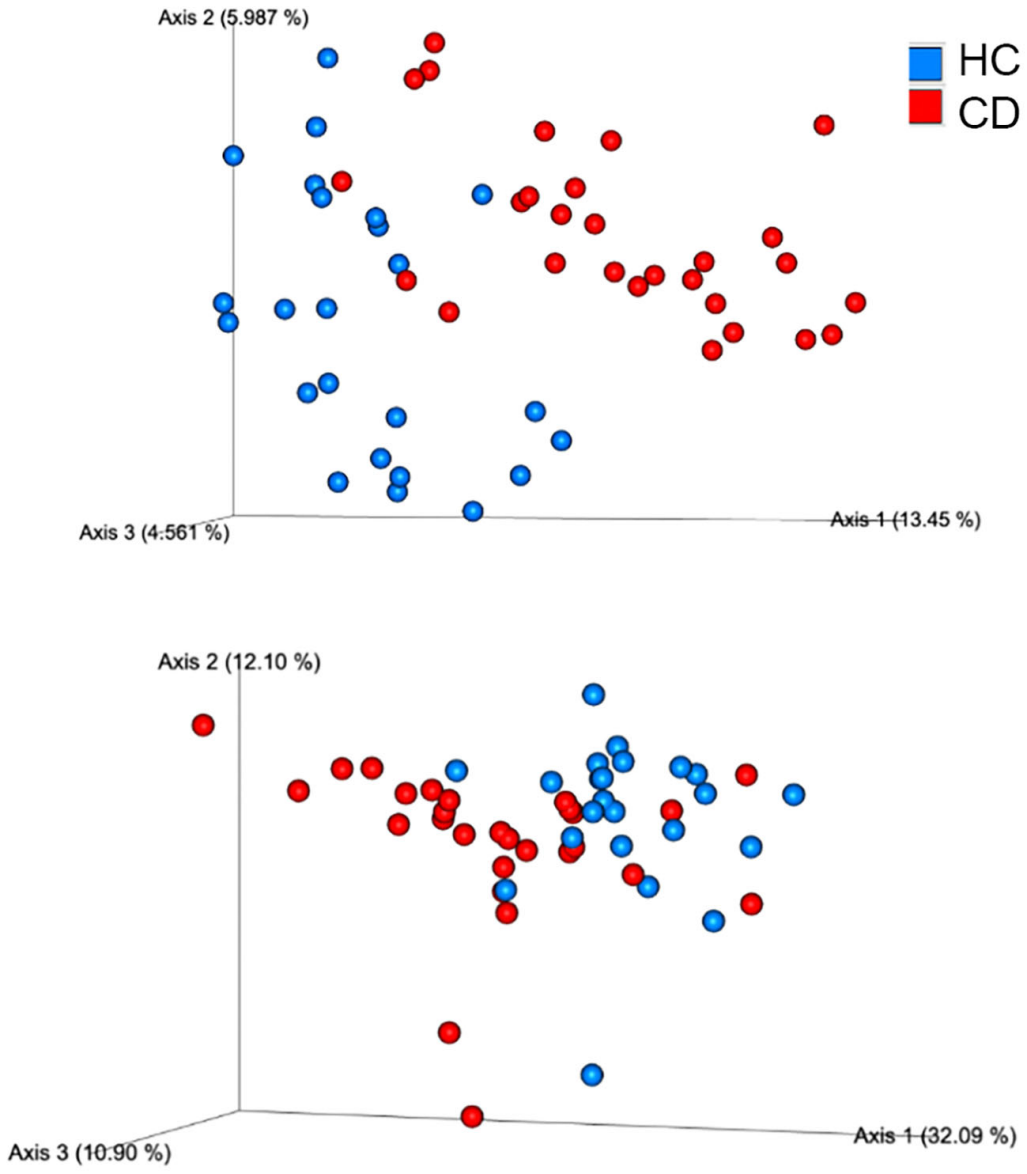
Beta diversity analysis, using the Principal Coordinates Analysis (PCoA) of weighted and unweighted UniFrac distances, showed that fecal samples from patients with Cushing’s disease (red circles) clustered together and separated from HC samples (blue circles). The plot is based on the distance analysis between samples. The closer the samples are to each other, the more similar their microbiome composition in terms of richness (for unweighted UniFrac metrics) and in terms of evenness, based on the most abundant taxa (for weighted UniFrac metrics).

Beta diversity analysis, using the Principal Coordinates Analysis (PCoA) of weighted and unweighted UniFrac distances, showed that faecal samples from patients with CD clustered together and separated from the samples of the controls (Adonis test, q=0.001 for both weighted and unweighted UniFrac) (**Figure 3**).

**Figure 4:**
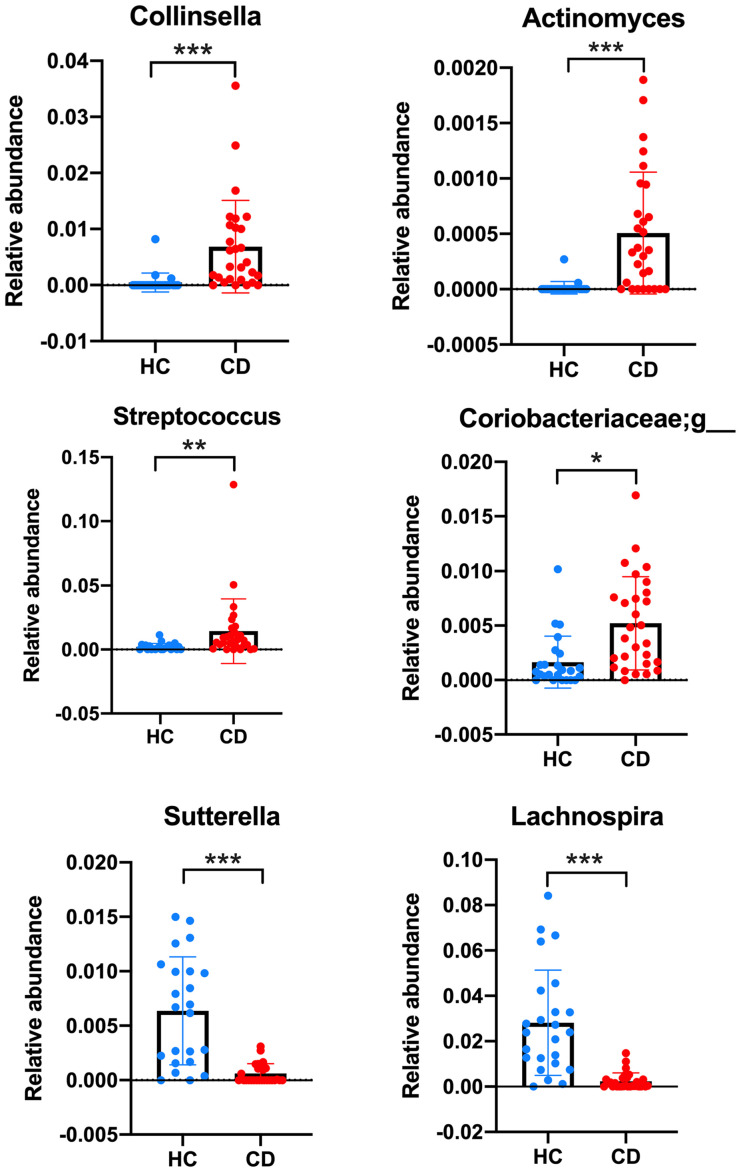
Bacterial genera significantly different between healthy controls (HC) and Cushing’s disease (CD). The relative abundance of bacterial genera was compared between HC and CD using the MaAsLin2 (Microbiome Multivariable Association with Linear Models) statistical tool, with medications and disease status as fixed effects and age and BMI as random effects. Corrected p-values were displayed: *q < 0.05; **q < 0.005; ***q < 0.005.

At the composition level, after adjusting for age, BMI and medication, the analysis showed that several GM groups at phylum, family, order, and genus were associated with CD as compared with control. *Collinsella, Actinomyces*, Streptococcus and an unknown genus from the Coriobacteriaceae were highly enriched in CD patients but almost absent in controls. *Sutterella* and *Lachnospira* were depleted in CD as compared with controls (**Figure 4**).

### Relationship between cardiometabolic parameters and gut microbiome (Chao 1 index)

In CD patients only, the Chao 1 index was associated with fibrinogen levels (ρ=0.44; p=0.034), and inversely correlated with both triglyceride concentrations (ρ=-0.48; p=0.035), glycemia (ρ=-0.42; p=0.044) and the HOMA-IR index (ρ=-0.45; p=0.038).

## Discussion

In this pilot study, we have demonstrated for the first time that patients with CD in long-term remission have significant gut dysbiosis, characterized by lower abundance and diversity of GM as compared with healthy controls. Patients also showed a preponderance of *Actinobacteria* over *Firmicutes*, with *Collinsella*, a genus from *Actinobacteria* phylum, being detectable in CD only. On the contrary, *Sutterella*, a genus from *Proteobacteria phylum*, was scarcely detectable/undetectable in CD patients as well as *Lachnospira*, a genus of the *Lachnospiraceae* family of the *Firmicutes* phylum. Moreover, we have shown that the Chao index, a marker of GM richness, was inversely associated with glycemia, HOMA-IR and triglycerides concentrations in CD patients only, suggesting that the sustained dysmetabolism in patients who have been previously exposed to high cortisol levels may be related to gut dysbiosis. Recent evidence has described that a disrupted GM may be a crucial factor in the pathophysiology of obesity, insulin-resistance, dyslipidemia, and cardiovascular disease in animal and human models (9). Several mechanisms whereby gut dysbiosis may contribute to the pathogenesis of metabolic syndrome have been described, and include altered choline and biliary acid metabolism, increased energy harvesting and utilization, and increased intestinal permeability (the “leaky gut”) (11). The latter is due to the impairment of the intestinal barrier induced by both inflammation and dysbiosis, which results in the translocation of bacterial pro-inflammatory products [endotoxins, short-chain fatty acids (SCFAs) and lipopolysaccharide (LPS)] into the circulation (Fan). These molecules usually activate an inflammatory cascade in liver, adipose tissue and muscle leading to insulin-resistance and sustained metabolic derangement (22). This results in a further deterioration of intestinal permeability, which generates a vicious circle perpetuating a chronic, systemic, low-grade inflammation state (22), as previously described to exist in treated CD (23). Infusion of acetate, a SCFA produced from the microbial fermentation of dietary fibres, altered insulin-sensitivity and glucose disposal, and increased intra-liver fat storage and plasma triglyceride content in rats (24). Injection of LPS in mice reduced HDL-cholesterol and increased plasma triglyceride concentrations, while a linear relationship has been documented between LPS levels and serum triglycerides, insulin-resistance and blood pressure in patients with type 2 diabetes (25, 26). Thus, GM plays a central role in the regulation of the inflammatory response that pathogenically links dysbiosis with obesity, insulin resistance and atherosclerosis. Interestingly, Zhang et al. recently showed that serum levels of propionic acid, one of the most abundant SCFAs, which is thought to promote favourable metabolic changes and protect against diet-induced obesity, were reduced in 23 patients with active CS as compared with controls (27). In agreement with our results, a relative scarcity of *Firmicutes* has also been documented in these patients, supporting our hypothesis that dysbiosis related to cortisol excess may persist long-term despite biochemical remission (27)

In agreement with previous studies, our “cured” non-diabetic CD patients were overweight and presented with higher percent fat mass, triglycerides and fibrinogen levels, and a greater degree of insulin-resistance, as compared with controls with similar BMI and waist circumference (3, 23). It has been previously shown that residual metabolic morbidity in CD patients was associated with an abnormal adipokine secretion profile which contributes to maintain a chronic low-grade inflammatory state (23). Thus, although the cross-sectional design of our studies prevents from inferring causality based on our data, it is intriguing to speculate that there is a profound interplay between GM and adipose tissue in “cured” CD patients, and gut dysbiosis is one of the mechanisms whereby the abnormal fat storage and consequent metabolic derangements typically found in active CD remain partially unchanged after remission. While future research is needed to elucidate if gut dysbiosis is associated with an altered cytokine profile in these patients, interventional studies should be carried out to investigate if the intake of probiotics or faecal microbiota transplants could improve the metabolic risk in CD as demonstrated in obesity (28–30).

Surprisingly, we have also found an association between high fibrinogen levels and the Chao index. A close pathogenic link exists between GM and thromboinflammation, in that the former promotes the formation, in the liver, of the pro-atherogenic molecule Trimethylamine N-oxide (TMAO) *via* the cleavage of dietary choline (31). We cannot infer from our data whether increased cardiovascular and atherosclerotic risk in CD may be related to gut dysbiosis (2). The correlation we have documented should be interpreted with caution and confirmed in future studies extensively assessing the coagulation profile in a large population of CD patients.

Interestingly, we have described abundance of the genus *Collinsella* in CD patients whereas it was not present in controls. *Collinsella* is the dominant taxon of the family *Coriobacteriaceae* and belongs to the phylum *Actinobacteria*. *Coriobacteriaceae* have been described to affect human metabolism through the impairment of intestinal cholesterol absorption, blunting of hepatic glycogenesis and induction of triglyceride synthesis (32). Abundance of *Collinsella* was associated with insulin levels in pregnant women regardless of BMI (33). Administration of *Collinsella* reduced the expression of tight junction proteins in enterocytes thus increasing gut leakage which led to metabolic endotoxemia in patients with rheumatoid arthritis (34). Increased levels of *Collinsella* were found in patients with type 2 diabetes mellitus and in patients with symptomatic atherosclerotic carotid artery stenosis (35, 36). A structured program of weight loss in obese patients determined an 8.4-fold reduction of the *Collinsella* abundance (37). Of note, low-fibre diet was inversely associated with *Collinsella* abundance, suggesting that a change in the daily intake of fibres may be encouraged in our patients in an attempt to revert this microbiome abnormality and improve the metabolic profile (32). Furthermore, we have shown that the abundance of *Sutterella*, a genus from *Proteobacteria*, is extremely low in CD patients. *Sutterella* has been described to decrease in individuals with depression as well as in obese patients with mood disorders (38, 39). To elucidate if the gut-brain axis is impaired in CD patients and could contribute to the well-known persistence of neuropsychiatric morbidity in them should represent an important goal of future research (40).

Limitations of this study include the small sample size and the lack of an extensive evaluation of the cytokine profile, which would have been useful to elucidate the mechanisms underlying the relationship between gut dysbiosis and persistent cardiometabolic risk in “cured” CD patients. An important strength of this preliminary report is that we have selected our sex-, age-, and BMI- -matched subjects in terms of dietary habits and use of medications which could interfere with GM composition, and also excluded subjects with intestinal disturbances and type 2 diabetes. Thus, our data have clearly shown a unique GM “signature” in CD patients which, if confirmed in larger studies, may provide an important diagnostic and therapeutic tool to assess and, possibly, treat the elevated cardiovascular risk associated with in this condition. Moreover, whether cortisol-lowering agents have the potential to induce favourable changes in GM characteristics is another interesting point that should be addressed in future studies.

In conclusion, we have demonstrated for the first time that patients with CD in long-term remission have gut microbial dysbiosis, with decreased microbiota richness and diversity, and specific variations in the bacterial community structure. While dysbiosis in CD may be one of the mechanisms whereby cardiometabolic dysfunctions persist after “cure”, future studies with a larger sample are needed to confirm our results and establish the potential benefits of specific interventions aimed at changing GM in CD patients with residual morbidity.

## Data availability statement

The data presented in the study are deposited in the NCBI repository, accession number PRJNA893885. 

## Ethics statement

The studies involving human participants were reviewed and approved by CEIC, Hospital Sant Pau, Barcelona. The patients/participants provided their written informed consent to participate in this study.

## Author contributions

EV conceived and designed the analysis, collected and analysed the data, wrote the paper. CM conceived and planned the experiments, analysed the data and contributed to write the paper. VA collected the data and contributed to the interpretation of the results. PF collected the data and contributed to the interpretation of the results. SG collected the data and contributed to the interpretation of the results. FY contributed to sample preparation and interpretation of the results. LM-D collected the data and contributed to the interpretation of the results. MP-D contributed to the interpretation of the results. SW collected the data and contributed to the interpretation of the results. All authors provided critical feedback and helped shape the research, analysis and manuscript. All authors contributed to the article and approved the submitted version.
